# Strangulated necrotic gastric fundus due to diaphragmatic hernia presenting with hydropneumothorax 5 years after left hepatic lobectomy: a case report and literature review

**DOI:** 10.3389/fsurg.2026.1802346

**Published:** 2026-05-21

**Authors:** Yibing Zhu, Qingyu Wang, Jingling Lai, Yinggang Qin, Li Sun, Ming Ye, Junyi Li

**Affiliations:** 1 Department of Emergency, Guang'anmen Hospital, China Academy of Chinese Medical Sciences, Beijing, China; 2 Department of Oncology, Guang'anmen Hospital, China Academy of Chinese Medical Sciences, Beijing, China; 3 Department of Urology, Guang'anmen Hospital, China Academy of Chinese Medical Sciences, Beijing, China; 4 Department of Radiology, Guang'anmen Hospital, China Academy of Chinese Medical Sciences, Beijing, China; 5 Department of Nephrology, Guang'anmen Hospital, China Academy of Chinese Medical Sciences, Beijing, China; 6 Department of Surgery, Guang'anmen Hospital, China Academy of Chinese Medical Sciences, Beijing, China

**Keywords:** case report, diaphragmatic hernia, hydropneumothorax, post-hepatectomy complication, strangulated gastric necrosis

## Abstract

**Introduction:**

Diaphragmatic hernia (DH) is characterized by the protrusion of abdominal organs into the thoracic cavity through a diaphragmatic defect. Acquired DH typically results from trauma, prior surgery, or increased intra-abdominal pressure. Diagnosing DH complicated by hydropneumothorax is challenging due to atypical initial symptoms, often leading to misdiagnosis as primary pulmonary conditions.

**Case description:**

A 60-year-old male presented to the emergency department with persistent respiratory distress, dyspnea, and an occasional cough. He had a surgical history of left hepatic lobe hemangioma resection five years prior. Emergency chest computed tomography (CT) revealed left-sided hydropneumothorax and herniation of the gastric fundus into the thoracic cavity. Emergency surgery confirmed necrotic gastric fundus caused by incarcerated diaphragmatic hernia. The patient underwent successful surgical repair and partial gastrectomy, leading to a full recovery.

**Conclusions:**

Acquired DH in adults can be insidious, with non-specific symptoms that mimic common respiratory or cardiovascular diseases. A critical and life-threatening complication is strangulated DH leading to gastric necrosis. Clinicians must maintain a high index of suspicion for DH in patients with unexplained chest tightness and shortness of breath who have a history of regional surgery, even years after the initial procedure.

## Introduction

Diaphragmatic hernia (DH) involves the translocation of abdominal viscera into the thoracic cavity via a defect in the diaphragm. While congenital forms exist, acquired DH is predominantly caused by blunt or penetrating trauma, surgical interventions, or chronic elevations in intra-abdominal pressure (1). This anatomical displacement can cause severe complications due to the compression of intrathoracic structures (2, 3).

Hydro-pneumothorax is a recognized complication of diaphragmatic hernia, resulting from disruption of the normal pleural environment when abdominal organs, such as the stomach, herniate into the thoracic cavity through a diaphragmatic defect. Strangulation and necrosis of the stomach secondary to incarcerated diaphragmatic hernia represent a severe, life-threatening complication (2, 3). However, diagnosis may be challenging because the presenting symptoms are often nonspecific and can mimic common respiratory or cardiovascular conditions, including chronic obstructive pulmonary disease, pneumonia, heart failure, and angina pectoris (4, 5).

According to previous studies, the incidence of post-hepatectomy diaphragmatic hernia is approximately 0.75%, with right hepatectomy being the most commonly associated surgical procedure (6–15). Reports of diaphragmatic hernia following resection of a hemangioma in the left hepatic lobe remain limited (6). The initial clinical manifestations may resemble those of common respiratory or cardiovascular diseases, such as chronic obstructive pulmonary disease, pneumonia, heart failure, and angina pectoris, which can contribute to delayed diagnosis. Given the rarity and complexity of this condition, as well as the potential involvement of multiple organ systems, reporting individual cases remains important to expand the current body of knowledge and support improved clinical management.

## Case description

A 60-year-old male was admitted to the emergency department with one week of respiratory distress, presenting with persistent chest tightness, dyspnea and occasional dry cough. The onset was unprovoked, and no history of chronic conditions including hypertension, diabetes, chronic obstructive pulmonary disease (COPD), or cardiocerebrovascular diseases was noted. A history of smoking and alcohol consumption was also denied. Notably, the patient had a surgical resection for a left hepatic lobe hemangioma 5 years prior to admission. The procedure was performed via a laparoscopic approach. The patient presented with multiple hepatic hemangiomas confined to the left hepatic lobe, with the largest lesion measuring approximately 7 cm in maximum diameter. No adhesion was noted between the lesions and the left hemidiaphragm. No diaphragmatic injury or repair was noted during the laparoscopic procedure, and no retractors were used intraoperatively. Within one week of symptom onset, no vomiting, retching, heavy physical labor, or strenuous exercise was reported; chest tightness was mild initially and progressed gradually, with no obvious factors for relief or aggravation. The patient presented to the hospital due to progressively worsening dyspnea on the morning of admission.

On admission, tachycardia (heart rate 113 beats per minute), increased respiratory rate (respiratory rate 20 breaths per minute), and mildly reduced peripheral oxygen saturation [peripheral oxygen saturation (SpO_2_) approximately 92% on room air] were identified. A semi-recumbent position was immediately adopted for the patient, with continuous cardiac and respiratory monitoring initiated and oxygen therapy administered via a nasal cannula at a flow rate of 3 L/min; SpO_2_ was maintained at 96%–99% following oxygen supplementation. Physical examination revealed right tracheal deviation, absent breath sounds over the left lung field, and clear breath sounds over the right lung field without auscultated rales, rhonchi or pleural friction rub. The abdominal wall was soft, with no tenderness, rebound tenderness or distension noted in the entire abdomen; bowel sounds were normal, and no palpable masses or hepatosplenomegaly were identified. Based on clinical symptoms and physical examination, initial diagnostic considerations included pneumonia, spontaneous pneumothorax, acute heart failure, and acute exacerbation of COPD. Routine blood testing, arterial blood gas analysis, and bedside chest x-ray (CXR) were therefore performed immediately.

**Figure 1:**
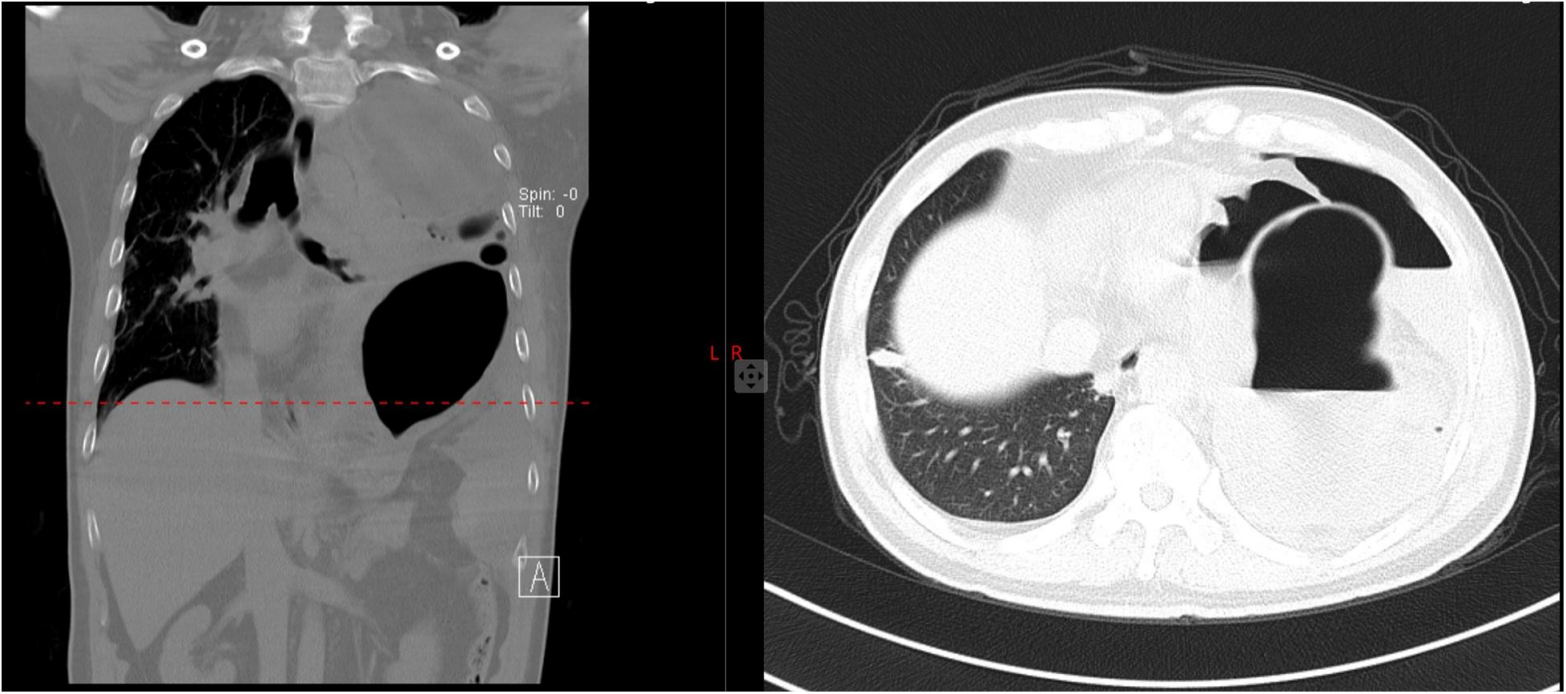
Chest CT plain scan: the left side is a coronal image, and the right side is an axial image (corresponding to the red dashed layer of the coronal position). The images show: a gas-containing gastric cavity is herniated into the left thoracic cavity (round-shaped hyperlucent shadow in the left thoracic cavity on the right axial image); the left lung tissue is compressed and collapsed (partial aggregation of lung markings can be seen in the right lung field); left pneumothorax is formed (a low-density area without lung markings in the left thoracic cavity), accompanied by mediastinal shift to the right.

Routine blood test results showed a white blood cell count of 14 × 10⁹/L, a neutrophil ratio of 79%, and a C-reactive protein (CRP) level of 165 mg/L. Arterial blood gas analysis on room air revealed a pH of 7.34, an arterial partial pressure of oxygen (PaO₂) of 66 mmHg, an arterial partial pressure of carbon dioxide (PaCO₂) of 35 mmHg, and a serum lactate level of 5.4 mmol/L. These indicators suggested the presence of infection, an inflammatory response, and tissue ischemia-hypoxia. CXR demonstrated right deviation of the mediastinum and trachea, decreased lucency of the left lung with indistinct lung markings, a lucent area in the left lung field, an elevated left hemidiaphragm with a visible gastric bubble, with the imaging impression of left hydropneumothorax complicated by left atelectasis. Immediate chest computed tomography (CT) was subsequently performed, which revealed left pneumothorax, left atelectasis, right mediastinal displacement, and herniation of the gastric fundus above the diaphragm with visible gas accumulation (Figure 1). DH with gastric herniation and pulmonary compression was diagnosed based on CT imaging findings.

Urgent chest exploration and transthoracic DH repair were then performed. Intraoperatively, the diaphragmatic defect was identified at the left hemidiaphragm, adjacent to the bare area of the left hepatic lobe, measuring approximately 5 cm in greatest dimension, with a concomitant DH measuring approximately 8 cm × 8 cm × 6 cm. Inflammatory exudate was noted around the hernia sac secondary to gastric herniation into the thoracic cavity, alongside a large volume of purulent secretion in the thoracic cavity, pulmonary congestion, and atelectasis. Upon incision of the diaphragmatic hernia sac, ischemic necrosis of the gastric fundus and gastric wall was confirmed.

Accordingly, combined DH repair and partial gastrectomy were performed. The diaphragm was incised to the esophageal hiatus for gastric repositioning into the abdominal cavity. The necrotic tissue was strictly confined to the gastric fundus, and resection was performed at a safe distance from the gastroesophageal (GE) junction with sufficient normal tissue preserved adjacent to the cardia. Intraoperative gross inspection confirmed a clear demarcation between necrotic and viable gastric tissue, and resection was completed under direct vision without the use of a bougie or intraoperative endoscope. No involvement or injury to the GE junction was identified. The necrotic gastric wall was then excised, followed by placement of a gastrostomy tube. The diaphragmatic defect was then closed, with closed thoracic drainage, gastrostomy, and abdominal drainage tubes placed sequentially.

Postoperatively, the patient received antibiotic therapy, anticoagulation, proton pump inhibitor administration, and nutritional support. Pathological examination of the partially resected gastric tissue revealed active inflammation and ulceration. The patient was discharged 10 days after admission, following drainage tube removal and continuous gastrostomy tube clamping. Outpatient surgical follow-up was conducted two weeks after discharge, with the patient in good general condition. The gastrostomy tube was removed three months postoperatively.

## Discussion

Post-hepatectomy DH is a rare postoperative complication with an incidence of merely 0.75%, and right hepatectomy is the most frequently reported procedure associated with this complication (6–15). In contrast, cases of DH following left hepatic hemangioma resection, as observed in the present patient, are extremely scarce in the existing literature (6). This rarity, combined with the insidious onset and non-specific early clinical manifestations of acquired DH, often leads to delayed diagnosis or misdiagnosis in clinical practice. Gastric strangulation or obstruction due to diaphragmatic hernia can be lethal (6–15).

The patient in this case presented with chest tightness and dyspnea as the core clinical symptoms, which are highly overlapping with those of common respiratory and cardiovascular disorders, including chronic obstructive pulmonary disease, pneumonia, and heart failure (4, 5). Such symptom similarity easily diverts clinicians' attention from considering DH as a potential diagnosis, particularly when the interval between the original liver surgery and the onset of hernia symptoms is as long as 5 years. A review of 104 reported cases of post-hepatectomy DH (summarized in Table 1) reveals that the time from liver surgery to DH diagnosis ranges from 12 weeks to 6 years, with a median interval of several months to years. This prolonged latent period further increases the difficulty of early diagnosis, as clinicians may not readily correlate late-onset thoracoabdominal symptoms with a remote history of liver surgery.

**Table 1:** Summary case information on diaphragmatic hernia after hepatectomy.

Reference and publication time	Number of cases	Age	Gender	Etiology	Procedure	LS to DH	Reason of presentation	Diagnostic Study
Raakow J (7)，2021	5	C1：55；C2：56；C3：58；C4：49；C5：69	C1：male；C2–5：female	C1: CRLM; C2: CCC; C3: CRLM; C4: HCC; C5: Cholecystitis	All were extended RH	C1: 21 months; C2: 15 months; C3: 34 months; C4: 44 months; C5: 36 months	C1: Intestinal obstruction; C2: Dyspnea; Ca3: Colonic stenosis found during colonoscopy; C4: Recurrence of liver cancer detected during follow-up, with asymptomatic DH; C5: Enterothoracic fistula complicated by jejunal perforation and peritonitis	CT; C4 additionally used MRI
Kara S (14)，2022	9	40.89 ± 12.8	4 females, 5 males	5 cases were living donor hepatectomy (donor liver transplantation), 1 case was liver transplantation, and 3 cases were hepatic alveolar echinococcosis	5 cases were LDLT donor hepatectomy, 1 case was liver transplantation, and 3 cases were RH	47.33 ± 38.16 months	1 case of abdominal pain, 5 cases of intestinal obstruction, 1 case of nausea, 2 cases of dyspnea or empyema	CT
Takaichi S (18)，2018	1	30	male	trauma	RH	5 years	Intestinal obstruction	CT
Yonemura Y (19)，2013	1	81	male	giant hepatic hemangioma	RH	20 months	nausea, abdominal pain	CT
Martin V (20)，2021	18 (13 adults, 5 children)	Adults: 50 (30–67) years old; Children: 2.4 (0.9–4) years old	Adults: 6 females, 7 males; Children: 2 females, 3 males	Adults: 5 cases of donor hepatectomy, 1 case of total LT, 2 cases of HCC, 1 case of giant hemangioma, 1 case of hepatic adenomatosis, 1 case of epithelioid hemangioendothelioma, 1 case of polycystic liver disease; Children: 2 cases of biliary atresia, 1 case of progressive familial intrahepatic cholestasis, 1 case of Alagille syndrome, 1 case of Budd-Chiari syndrome	Adults: 5 cases of right hepatectomy for living donor LDLT, 2 cases of whole liver transplantation, 5 cases of right hepatectomy related to tumors, 1 case of left LH; Children: 2 cases of left lateral segmentectomy for LDLT, 3 cases of left lateral segmentectomy for split LT	Adults: 65.1 (1.8–244.7) months; Children: 2 (0.33–10.9) months	Adults: 7 cases of abdominal pain, 3 cases of chest symptoms, 3 cases of dyspnea, 2 cases of intestinal obstruction, 6 cases of accidental discovery (asymptomatic); Children: 2 cases of abdominal pain, 2 cases of chest symptoms, 2 cases of pleural effusion, 2 cases of dyspnea, 2 cases of intestinal obstruction, 2 cases of fever, 1 case of intestinal perforation, 1 case of accidental discovery (asymptomatic)	Adults: CT, X—ray; Children: CT, X—ray. One case of digestive fluid drained from the thoracic drainage tube after liver transplantation, and surgical exploration was performed directly without CT.
Kim HJ, (21) 2021	6	NA	NA	LD	Right hepatectomy	4.95 （0.24–17.00) years	All presented abdominal pain as the main clinical manifestation	CT
Lee SW (8), 2021	1	61	male	Liver cancer	RH	1 year	Four days before being found dead, he complained of severe pain in his right chest.	autopsy
Kawada J (22), 2020	1	70	female	Liver cancer	LH	9 months	Epigastric pain	contrast-enhanced CT
Korkut E (23), 2019	1	28	female	Hepatic alveolar echinococcosis	RH	20 months	Abdominal pain, nausea and vomiting, respiratory distress	X-ray and CT
Lochan R (24), 2017	1	young, specific age unknown	male	LD	Right hepatectomy	12 weeks	Severe and persistent abdominal pain	CT
Esposito F (10), 2017	3	55 （43–65）	2 females, 1 male	2 cases of hepatocellular carcinoma, 1 mucinous cystadenoma	Right hepatectomy	14（4–31） months	abdominal pain in 1 case, asymptomatic in 2 cases	CT and MRI
Oh JW (25), 2017	9	46.0（12.85）	6 males, 3 females	LD	RH	173（range： 98–488 ） days	abdominal pain in 2 cases, asymptomatic in 7 cases, detected during routine postoperative CT follow-up	CT
Livingstone SM (26), 2016	2	25 and 23	1 female and 1 male	LD	RH	5 years and 19 months	abdominal pain, nausea, vomiting in C1, nausea, vomiting, severe abdominal pain and chest pain in C2	X-ray and CT
Patrizi A (27), 2016	3	NA	NA	NA	Hepatectomy	18 months in 1 case, unknown in 2 cases	Bilothorax in 2 cases, difficulty in breathing in 1 case	CT
Jeng KS (28), 2015	1	42	female	LD	RH	20 days	Severe upper abdominal pain, back pain, and cold sweats	CT
Lodhia JV (9), 2015	1	65	female	Liver metastases	LH	9 months	asymptomatic	PET-CT
Mizuno S (29), 2014	1	43	male	LD	LH	34 months	Upper abdominal pain, nausea, vomiting	CT
Vernadakis S (30), 2012	1	46	female	LD	RH	2.5 years	Upper abdominal pain, nausea, vomiting	CT
Tabrizian P (11), 2012	10	48.5 ± 14.4	5 males, 5 females	LD in 1 case, liver cancer in 9 cases	RH in 8 cases, left lateral segmentectomy of the liver, LH in 1 case	15 (r5–120) months	Abdominal pain or intestinal obstruction in 8 cases, asymptomatic in 2 cases	CT
Kim SH (31), 2025	18	NA	NA	LD	Right-sided living donor hepatectomy in 17 cases, left-sided living donor hepatectomy in 1 case	11 (3–95) months	Abdominal pain or intestinal obstruction in 8 cases, asymptomatic in 10 cases	NA
Dieter RA Jr (32), 2011	2	NA	NA	LD	RH	36 months	Abdominal pain and respiratory symptoms	NA
Kousoulas L (33), 2011	2	NA	NA	LD	RH	NA	NA	NA
Perwaiz A (34), 2010	1	44	male	End-stage cryptogenic liver disease complicated with recurrent spontaneous bacterial peritonitis and hepatic encephalopathy, undergoing living-related left liver lobe transplantation	LH	28 months	Abdominal pain	CT
Schellhaas P (13), 2010	1	54	female	Hepatic hemangioma	RH	6 years	Respiratory symptoms	X-ray
Matz D (35), 2009	1	67	male	Liver metastasis of sigmoid colon cancer	RH	3 years	Abdominal pain	X-ray and CT
Hawxby AM (12), 2006	1	54	male	LD	RH	3 years	Upper abdominal pain and a feeling of fullness	CT
Sugita M (15), 2003	1	31	female	Focal Nodular Hyperplasia	LH	8 months	Abdominal pain	MRI
Kim HC (36), 2012	1	26	male	LD	RH	3 months	Upper abdominal pain and vomiting	CT
Hemming AW (37), 2002	1	NA	NA	Malignancy	Hepatectomy	3 months	Sepsis	NA

C, case; CCC, cholangiocarcinoma; CRLM, colorectal liver metastases; CT, computed tomography; DH, diaphragmatic hernia; HCC, hepatocellular carcinoma; LD, liver donation; LDLT, living donor liver transplantation; LH, left hepatectomy; LS, liver surgery; LT, liver transplantation; MRI, magnetic resonance imaging; NA, not applicable (data not reported in the original study); RH, right hepatectomy. For reports containing multiple cases, data presentation was based on the results reported in the original article. Age and the time interval from liver surgery to the diagnosis of DH may be presented as mean ± standard deviation or median (range) in the table.

CT is an irreplaceable diagnostic modality for post-hepatectomy DH and serves as the gold standard for diagnosing complicated DH (16). In this case, chest CT clearly demonstrated herniation of the gastric fundus into the thoracic cavity, left pneumothorax, and mediastinal displacement, providing direct and definitive evidence for confirming the diagnosis and formulating an individualized surgical plan. For medical institutions with limited access to CT, chest x-ray (CXR) and bedside ultrasound are recommended as first-line preliminary imaging modalities for initial screening. Once DH is suspected based on clinical manifestations and preliminary imaging findings, patients should be promptly referred to a multidisciplinary specialist team consisting of general surgery and thoracic surgery clinicians for further evaluation and decision-making regarding surgical intervention. For patients with severe respiratory distress or hemodynamic instability, emergency consultation with critical care medicine specialists is prioritized to provide adequate respiratory and hemodynamic support before specialist referral.

The pathogenesis of post-hepatectomy DH is multifactorial and involves a combination of surgical and physiological factors. On one hand, surgical trauma incurred during hepatectomy may cause subtle, subclinical damage to diaphragmatic tissue; over time, the gradual weakening of diaphragmatic structural integrity at the injured site can lead to the formation of diaphragmatic defects. On the other hand, postoperative elevations in intra-abdominal pressure—caused by factors such as constipation, persistent coughing, or heavy physical activity—can act as a precipitating factor, promoting the protrusion of abdominal organs through pre-existing diaphragmatic defects and ultimately leading to hernia formation (1–3). Notably, the patient in this case had no clear history of acute intra-abdominal pressure elevation before symptom onset, suggesting that surgical trauma-induced structural weakness of the left diaphragm may be the primary pathogenic factor for DH development. Anatomical characteristics also play a role in the lateralization of post-hepatectomy DH: blunt or penetrating traumatic DH is predominantly left-sided, attributed to the protective effect of the right hepatic lobe against right diaphragmatic injury (17). However, in post-hepatectomy settings, right hepatectomy is more frequently associated with DH (as shown in Table 1), likely due to the larger surgical field and greater potential for iatrogenic diaphragmatic disturbance during right hepatic resection. The occurrence of left-sided DH in this patient following left hepatectomy further confirms that ipsilateral diaphragmatic surgical trauma is a key risk factor for post-hepatectomy DH, regardless of the surgical side of hepatectomy.

A comprehensive review of 29 published studies encompassing 104 cases of post-hepatectomy DH (Table 1) (7, 9–14, 18–37) identifies several notable clinical patterns and characteristics of this complication. In terms of etiology, liver donation for living donor liver transplantation and hepatocellular carcinoma resection are the most common indications for hepatectomy leading to subsequent DH, accounting for a substantial proportion of reported cases. Regarding clinical manifestations, abdominal pain and intestinal obstruction are the most frequent presenting symptoms, while a considerable number of patients are asymptomatic and diagnosed incidentally during routine postoperative follow-up imaging. This finding indicates that post-hepatectomy DH exhibits highly diverse clinical manifestations, and asymptomatic cases may be significantly underdiagnosed in clinical practice. The World Society of Emergency Surgery (WSES) Position Paper on complicated diaphragmatic hernia emphasizes that surgical repair is the definitive treatment for symptomatic DH or complicated cases, while conservative management with close monitoring is reserved for asymptomatic, small defects (16). For patients with thoracic and abdominal symptoms after hepatectomy, DH should be suspected, and imaging examinations (CT is preferred) should be performed in a timely manner to confirm the diagnosis.

This case report has certain inherent limitations that warrant acknowledgment. First, intraoperative imaging records of the diaphragmatic defect and hernia sac were not obtained, which limits the detailed characterization of the anatomical abnormality at the time of surgery. This deficiency has prompted the establishment of standardized intraoperative recording procedures in our clinical practice to optimize the completeness of clinical data and the methodological quality of future case reports. Second, the primary pathogenic factor for DH in this patient is inferred based on clinical evidence and exclusion of other contributing factors, and no direct objective experimental evidence has been obtained to confirm that surgical trauma-induced diaphragmatic structural weakness is the definitive cause of the diaphragmatic defect and subsequent hernia formation. Further basic and clinical research is needed to elucidate the precise mechanisms underlying the development of post-hepatectomy DH.

## Conclusions

This case highlights that post-hepatectomy DH, although rare, exhibits distinct clinical characteristics: insidious onset, atypical clinical symptoms, a long latent period, and a tendency to be misdiagnosed. Clinicians should enhance their awareness of this complication, especially for patients with a history of thoracoabdominal surgery who present with unexplained chest tightness, dyspnea, abdominal pain, or intestinal obstruction symptoms. Timely chest and abdominal CT is crucial for early diagnosis. Once diagnosed, surgical repair should be performed promptly to relieve organ compression, correct anatomical abnormalities, and avert serious complications including visceral necrosis and infection. The surgical approach and intraoperative management strategies should be individualized in accordance with the patient's clinical status and associated complications. Furthermore, postoperative long-term follow-up is essential for surveillance of recurrence and late-onset complications.

## Data Availability

The original contributions presented in the study are included in the article/Supplementary Material, further inquiries can be directed to the corresponding authors.
